# SNapp, a Tailored Smartphone App Intervention to Promote Walking in Adults of Low Socioeconomic Position: Development and Qualitative Pilot Study

**DOI:** 10.2196/40851

**Published:** 2023-04-17

**Authors:** Anne L Vos, Gert-Jan de Bruijn, Michel C A Klein, Jeroen Lakerveld, Sophie C Boerman, Edith G Smit

**Affiliations:** 1 Amsterdam School of Communication Research University of Amsterdam Amsterdam Netherlands; 2 Department of Communication Studies University of Antwerp Antwerp Belgium; 3 Social Artificial Intelligence Group Department of Computer Science Vrije Universiteit Amsterdam Amsterdam Netherlands; 4 Epidemiology and Data Science Amsterdam University Medical Centers Location Vrije Universiteit Amsterdam Amsterdam Netherlands; 5 Strategic Communication Group Wageningen University & Research Wageningen Netherlands

**Keywords:** cardiovascular disease, physical activity, walking, smartphone, mobile health, mHealth, mobile app, behavior change techniques, tailoring, intervention development, socioeconomic position, mobile phone

## Abstract

**Background:**

Adults of low socioeconomic position (SEP) are generally less physically active than those who are more socioeconomically advantaged, which increases their cardiovascular disease incidence risk. Moreover, individuals of low SEP are often less easily reached with physical activity (PA) interventions than individuals of higher SEP. Smartphone apps have been presented as a promising platform for delivering PA interventions to difficult-to-reach individuals of low SEP. Although PA apps are widely available, they are rarely based on health behavior theories and most predominantly offer generic PA advice. Consequently, it is unlikely that available apps are the most effective PA intervention tools.

**Objective:**

To respond to these areas for improvement, we developed SNapp, an app-based intervention encouraging adults of low SEP to increase PA by providing tailored coaching messages targeting walking behavior. This study aimed to describe SNapp’s stepwise development and pilot evaluation process.

**Methods:**

We applied a stepwise approach: analyzing the health problem, developing a program framework, developing tailoring assessments, writing tailored messages, automating the tailoring process, and implementing and evaluating the program in a qualitative pilot study (11 participants).

**Results:**

SNapp consisted of several elements. First, an app was developed to collect step count and geolocation data using smartphone sensor functionalities. In addition, a survey measure was created to assess users’ behavior change technique (BCT) preferences. These 3 data types were used to tailor SNapp’s coaching messages to stimulate walking. This allows SNapp to offer feedback on performance levels, contextually tailored prompts when users are near green spaces, and coaching content that aligns with individual BCT preferences. Finally, a server-based Python program that interacts with databases containing user data and tailored messages was built using Microsoft Azure to select and automatically send messages to users through Telegram messenger. Pilot study findings indicated that SNapp was rated positively, with participants reporting that its design, technical functioning, and message content were acceptable. Participants suggested additional functionalities that are worth considering for future updates.

**Conclusions:**

SNapp is an app-based intervention that aims to promote walking in adults of low SEP by offering tailored coaching messages. Its development is theory based, and it is among the first to incorporate contextualized feedback and content tailored to individual BCT preferences. The effectiveness of SNapp will be evaluated in a 12-month real-life parallel cluster-randomized controlled trial.

## Introduction

### Background

Cardiovascular disease (CVD) causes over 17 million deaths annually, making it the leading cause of death worldwide [1]. CVD is particularly prevalent among individuals of lower socioeconomic position (SEP), with research indicating that lower SEP groups have higher CVD mortality than more socioeconomically advantaged groups [2,3]. Physical inactivity is an essential modifiable CVD risk factor [4]. However, more than one-fourth (27.5%) of the adult population is insufficiently active for improving overall health and mitigating the risk of adverse health outcomes [5], including CVD incidence and mortality [6,7]. Furthermore, there is evidence that SEP is an important correlate of physical activity (PA) [8], indicating that adults of low SEP are less physically active than those of higher SEP [9,10]. Therefore, there is an urgent need for effective interventions encouraging individuals of lower SEP to become and remain physically active to reduce CVD risk.

Individuals of low SEP are often less easily reached with PA interventions than individuals of higher SEP [11,12]. With access to and sophistication of digital technologies such as mobile health apps increasing worldwide, smartphones have been presented as a promising platform to deliver PA interventions in a relatively cost-effective way to otherwise difficult-to-reach individuals of low SEP on a large scale [13,14]. Smartphones can unobtrusively collect information via built-in sensors, such as step count or distance moved, which apps can use to provide individually tailored support to improve PA levels. Given their increasing availability and popularity [15,16], PA apps have great potential to reach a large proportion of the population and create new opportunities for PA promotion.

Although PA apps are already widely available and seem promising for improving PA behavior [17], studies indicate that they are rarely based on health behavior theories [18,19], and most apps do not incorporate evidence-based behavior change techniques (BCTs; eg, goal setting, action planning, or self-monitoring) [20,21] or adhere to PA guidelines [22,23]. Health interventions are most effective in the long term when developed using a theoretical basis and if multiple BCTs are included [24,25]. In addition, systematic reviews have established that the effectiveness of PA interventions is enhanced when using individually tailored approaches [24,26] and providing context-specific feedback to improve PA performance levels [27,28]. However, most available apps predominantly offer generic PA advice, and contextualized feedback is rarely implemented [29]. Consequently, it is unlikely that these apps are the most effective intervention tools for promoting PA.

### Objectives

We developed an app-based PA intervention called SNapp to respond to these areas for improvement. SNapp is embedded in the larger Supreme Nudge project, aiming to improve lifestyle behaviors and cardiometabolic health in adults of low SEP by combining nudging and pricing strategies in a supermarket intervention with tailored PA coaching through a mobile app. A description of the relevance and design of the project has been published elsewhere [30,31]. With SNapp, we specifically aim to motivate adults of low SEP to increase activity levels by offering tailored support targeting walking behavior. Daily step count is a feasible measure for monitoring and motivating walking behavior that has been related to mortality benefits [32]. Within SNapp, smartphone sensor functionalities are combined with theory-based BCTs [33] to provide tailored coaching messages to stimulate walking. The content of these messages is individually tailored using step count, geolocation, and BCT preferences data.

In this paper, we describe SNapp’s stepwise development process, guided by the program planning model of Kreuter et al [34]. We aim to allow others to learn from our experiences and accelerate the development of similar app-based interventions, thereby aiding in mobile health intervention research advancement.

## Methods

### Development Process

The development of SNapp was guided by the program planning model of Kreuter et al [34]. Some steps from the original model were merged to fit our needs, resulting in a 6-step development process (Table 1). The development methods for each step are described in the following paragraphs of the *Methods* section. The results of each of the 6 steps are described in the *Results* section.

**Table 1:** The stepwise development process of SNapp (adapted from the program planning model of Kreuter et al [34]).

Steps	Step description
1. Analyzing the health problem	Identifying the determinants of walking behavior change Selecting behavior change techniques to address these determinants
2. Developing a program framework	Defining SNapp’s objectives Describing SNapp’s framework components
3. Developing tailoring assessments	Developing tailoring assessment questions to measure behavior change technique preferences Designing and developing an app for Android and iOS to collect step count and geolocation data
4. Writing tailored messages^a^	Creating a tailored message database Testing messages for readability to fit the literacy levels of the target population
5. Automating the tailoring process^b^	Storing user data Developing tailoring algorithms that automatically link user data with tailored messages Creating a communication channel to deliver tailored messages
6. Implementing and evaluating the program^c^	Pilot testing to detect bugs and assess the usability of SNapp

^a^This step combines steps 4 (“designing feedback”) and 5 (“writing tailored messages”) of Kreuter et al [34].

^b^This step combines steps 6 (“creating tailoring algorithms”) and 7 (“automating the tailoring process”) of Kreuter et al [34].

^c^This step combines steps 8 (“implementing the program”) and 9 (“evaluating the program”) of Kreuter et al [34].

### Step 1: Analyzing the Health Problem

#### Identifying Determinants of Behavior Change

The health problem that SNapp aims to address is physical inactivity, thereby reducing CVD risk. The target population was adults of low SEP aged 45 to 75 years. Low SEP was defined as having a practical vocational or lower education level and living in a low SEP neighborhood. SNapp focuses on 1 specific behavior to enhance PA in this population: walking. Improving daily activity levels through light to moderate intensity PA, such as walking, is favorably associated with health benefits, including cardiometabolic risk factors [35,36].

We reviewed applicable health behavior theories and previous research to identify the main determinants influencing the target group’s walking behavior [37]. A previously implemented app-based PA intervention that used a similar theoretical foundation is the Active2Gether intervention. The Active2Gether app was developed to increase sports participation, stair climbing, and active transport (for a detailed description of this intervention, refer to the studies by Klein et al [38] and Middelweerd et al [39]). Elements of this app, including its underlying theoretical framework and behavioral determinants, were taken as a basis to inspire SNapp’s development and adapted to fit our distinct PA behavior.

#### Selecting BCTs

Although determinants may help identify the mechanisms for behavior change, there is still the need to select appropriate strategies to change them. To operationalize the selected determinants, we consulted previous research and the literature on BCTs [40]. The evidence-based BCTs used in the Active2Gether intervention [39] were also relevant for SNapp. These techniques were selected from the BCT taxonomy for PA behavior change by Michie et al [40], based on the combined results from a content analysis of BCTs used in existing PA apps [19], focus group discussions [41], and a cross-sectional survey concerning adults’ tailoring preferences and ratings of the importance of specific BCTs in PA apps [42].

### Step 2: Developing a Program Framework

#### Defining Objectives

The second step was to create a program framework that served as a blueprint for SNapp. To achieve this, the objectives of SNapp were first defined. These objectives were informed by the World Health Organization’s PA guidelines for adults translated in terms of steps per day [43].

#### Describing Framework Components

**Figure 1:**
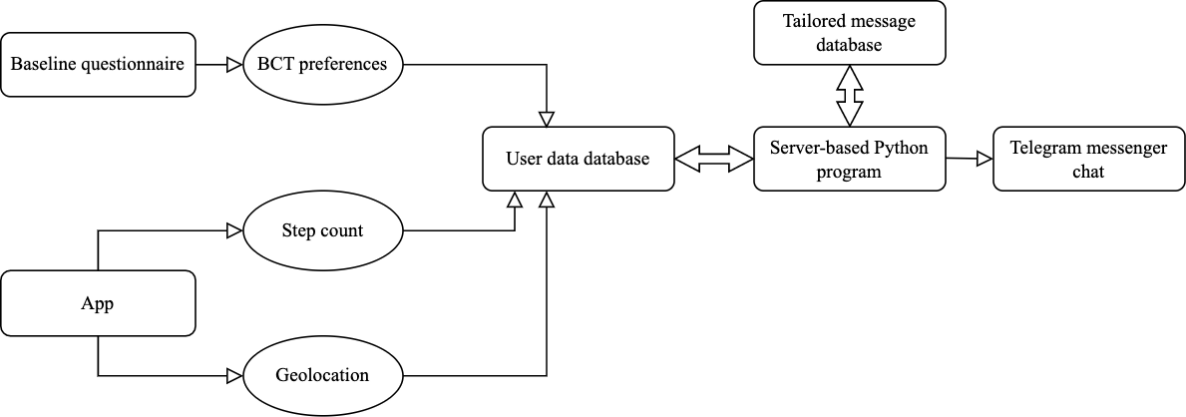
Components of the SNapp framework. BCT: behavior change technique.

Tailored health interventions consist of data collection and feedback delivery modules. In this development stage, we identified the most appropriate format, type, frequency, and timing of feedback delivery. In addition, we determined the data collection methods that should be used within the SNapp framework to facilitate the delivery of tailored feedback. An overview of the components that we strived to include within the SNapp framework is provided in Figure 1. The components are described in detail in the *Results* section. In short, it was first determined that an app would be developed to collect step count and geolocation data using smartphone sensor functionalities. In addition, a survey measure would be created to assess users’ BCT preferences. These 3 data types would be used to tailor coaching messages to stimulate walking. This would allow SNapp to offer highly tailored feedback on performance levels, prompts when users are near green spaces, and coaching content that aligns with individual BCT preferences. Finally, using Microsoft Azure, a server-based Python program that interacts with databases containing user data and tailored messages would be developed to select and automatically send messages to users through Telegram messenger.

### Step 3: Developing Tailoring Assessments

#### Developing Tailoring Assessment Questions

First, this step entailed creating a measure to assess BCT preferences using a web-based baseline questionnaire (Figure 1). Our objective was to develop a measure that would be easy to understand and brief to complete. This measure was formulated based on the BCT taxonomy by Michie et al [33].

#### Designing and Developing an App

Next, we formulated the requirements for developing our app (Figure 1). First, we aimed to build a robust, high-quality smartphone app that would perform optimally. It should track step count and geolocation continuously without user input or additional hardware use. In terms of functionalities for the user, we wanted it to display only the number of steps taken on the current day to ensure that no other app functionalities would influence walking behavior. We wanted the app to run on both Android and iOS devices and on high- and low-end smartphones to ensure usability for as many users as possible. We required the app’s development to be privacy aware, meaning that users would not need to provide personal information such as their telephone number or email address to use the app.

In addition, we determined that the app should not store or reveal the location of the user. Instead, we wanted the app to recognize whenever the device would be near a location included in a database of preselected green spaces, avoiding the need to repeatedly send the exact coordinates of the user’s location to the server. Regarding design and aesthetics, we strived to create a user-friendly app with an appealing interface for a positive user experience. A team of professional app developers was hired to ensure the development of a qualitatively strong app. To maximize the robustness of SNapp, we aimed to use existing smartphone services as much as possible. Therefore, we decided to use an existing messaging app to deliver tailored coaching messages.

### Step 4: Writing Tailored Messages

#### Creating a Tailored Message Database

The next step was to create a tailored message library. We reused parts of the previously conducted Active2Gether intervention’s message library [44]. As not all messages could be reused, as this intervention targeted other forms of PA, new messages in a similar style were added to supplement the message database. The aim was to write messages that addressed the user by name; had a friendly, positive, and encouraging tone; followed the format of mobile text messages; and contained approximately 20 to 30 words.

#### Testing Messages for Readability

To assess whether the reading level of the messages was appropriate for the target population, we used a Dutch web-based readability tool [45] that uses the reference levels of the Common European Framework of Reference for Languages to rate the complexity of written texts [46].

### Step 5: Automating the Tailoring Process

#### User Data Storage

Next, we started automating the tailoring process. This first involved setting up a database using Microsoft Azure to store user data regarding BCT preferences, step count, and geolocation (Figure 1).

#### Developing Tailoring Algorithms

Subsequently, we developed tailoring algorithms to automatically link user data with the tailored messages that users should receive. Within the SNapp framework, coaching messages are stored in a separate database with tailored messages. To select appropriate messages to send to users, we created a server-based Python program using Microsoft Azure that interacts with the databases containing user data and tailored messages (Figure 1). The program uses logical rules that are evaluated at specific times during the day to determine which message should be sent. In addition, restrictions on the circumstances under which messages are relevant are considered (eg, day of the week and time of the day).

#### Creating a Communication Channel

Subsequently, we selected an existing messaging service to deliver tailored coaching messages. We decided to use the Telegram messenger app to safeguard the robustness of SNapp. Telegram has several advantages. The messaging app provides data security for users, is fast and easy to use, and can be installed free of charge on both Android and iOS devices. Furthermore, it has an application programming interface service that developers can access for various needs. We subsequently created a SNapp Telegram account following the BotFather setup [47]. We received an application programming interface token that allowed our server-based Python program to communicate with the SNapp Telegram account to send tailored messages to users (Figure 1).

### Step 6: Implementing and Evaluating the Program

An exploratory pilot study was conducted among 11 users (9 female users, aged 54-69 years, and 7 Android users) to gain insight into the technical functioning and user evaluation of SNapp. Participants were recruited through community centers in Amsterdam, the personal network of the authors, and Facebook groups. Inclusion criteria included being ambulatory and aged ≥45 years; speaking the Dutch language; having experience with receiving mobile text messages; and using a smartphone with a mobile data plan and Android 8, iOS 13, or more recent software versions installed.

Participants used SNapp for 2 weeks and were interviewed at the end of each week during which they were asked several open questions by means of a topic list to gauge their opinion on the different components of the SNapp coaching system. Participants were encouraged to provide honest feedback about the app in terms of its usability and their overall experience. The interviews were conducted through Zoom videoconferencing (Zoom Video Communications) and lasted for approximately 30 minutes. Two researchers conducted the interviews, with one leading the interview and the other taking notes. The interviews were recorded and transcribed, and the notes were discussed and summarized. The transcripts and summaries were analyzed using grounded theory [48], based on a coding framework developed from the topic list of interview questions. Subsequently, all codes from the separate interviews were compared and organized to reveal the thematic categories.

### Ethics Approval, Informed Consent, and Participation

The Faculty Ethics Review Board of the University of Amsterdam approved all procedures (#2019-PC-11457). Participants provided written informed consent before participation. They were informed that their anonymity would be guaranteed and that their data would not be provided to third parties under any circumstances. In addition, they were informed that they could refuse to participate in the study or terminate their participation prematurely without providing a reason. They could also withdraw their consent for the use of their data for research purposes within 7 days of participation. The participants received a financial reward of €20 (US $24.51) after completing the pilot study.

## Results

### Step 1: Analyzing the Health Problem

#### Identifying Determinants of Behavior Change

Social cognitive theory (SCT) [49] was considered the most appropriate theoretical framework for the development of SNapp. SCT’s constructs are considered reliable correlates of PA [50,51], with a comprehensive meta-analytic review demonstrating that SCT accounts for approximately 31% of the variance in PA [37], which allows the theory to be considered a useful framework for PA intervention design according to the recommendations of Baranowski et al [52]. Moreover, the proportion of PA variance explained by SCT has been shown to increase for older samples [37], underscoring the usefulness of SCT for developing PA interventions for adults. Finally, there is evidence that SCT concepts improve the efficacy of tailored health interventions [53].

The 4 resulting SCT constructs selected as the main determinants to be addressed by SNapp are self-efficacy, outcome expectations, sociostructural factors, and goals (Table 2). Self-efficacy is defined as an individual’s beliefs concerning their ability to perform a desired behavior, such as engaging in PA. Outcome expectations are defined as an individual’s beliefs regarding the possible positive and negative consequences of performing the behavior. In addition to these 2 cognitions, the third core construct of SCT comprises sociostructural factors. These concern an individual’s perceptions of the barriers and opportunities that are present in their environment or life circumstances and that facilitate or hinder the desired behavior. Finally, SCT includes goals as a core determinant of behavior. SCT posits that before performing a desired behavior, individuals first need to set goals in terms of intentions to act to guide performance of the desired behavior [54].

**Table 2:** Correspondence between determinants, behavior change techniques, and their applications in SNapp.

Determinant and behavior change technique		Application in SNapp	Example
**Self-efficacy**			
	Action planning	Messages prompting users to plan their walks (eg, when, where, and with whom they will go for a walk in the upcoming week)	“Good preparation is half the battle won! Plan and write down when and where you will go for a walk in the coming days.”
	Reward	Messages praising users for attempting to engage in regular walking and encouraging them	“Hey <name>, keep up the good work! Try to keep walking every day. You can do it.”
	Feedback on performance	Messages providing data about step count levels and commenting on user performance	“So far you have taken <number> steps today, <name>. Well done! You can be proud of yourself.”
	Social comparison	Messages stating to what extent other SNapp users are reaching their walking goals	“Hi <name>, here’s a little fact: last week, 73% of SNapp users took enough steps every day!”
**Outcome expectations**			
	Provide information on the consequences of behavior	Messages providing information on the positive consequences of regular walking (eg, related to health, appearance, or mood)	“Did you know that walking can help relieve stress? Walking makes your brain release chemicals that stimulate relaxation and improve your mood.”
**Sociostructural factors**			
	Provide instruction	Messages informing users they are currently near a green space where they can walk	“Do you want to get some extra steps in today? You are close to a <*green space type*>where you can enjoy a nice walk.”
	Barrier identification	Messages identifying potential barriers to engaging in regular walking and ways of overcoming them	“No time to go for a walk? Get your steps in throughout the day by taking the stairs, getting off the bus a stop earlier, or parking further away.”
	Social support	Messages prompting users to tell others in their social circle about their walking goals and plan for support	“Hey <name>, have you told your friends or family about your walking goals? That way, they can support or join you for a walk!”
	Social approval	Messages informing users that others in their social circle and other SNapp users approve of them attempting to engage in regular walking	“Good morning <name>. If you manage to keep walking regularly, your friends and family will surely be proud of you.”
**Goals**			
	Goal setting	Messages encouraging users to set a daily step count goal for the upcoming week	“What’s your new goal, <name>? Set yourself an achievable walking goal for this week.”
	Self-monitoring	Messages prompting users to check the step counter app to keep track of their daily step count levels	“Curious to know how many steps you’ve already taken today? Try to keep track by checking the step counter every day.”
	Prompt review of outcome goals	Messages prompting users to review to what extent they achieved their walking goals	“Did you achieve this week’s walking goal, <name>? If so, good job! If not, try to think of a new goal for next week.”

#### Selecting BCTs

On the basis of the study by Michie et al [40,55], suitable BCTs were subsequently selected and linked with the determinants to be addressed by SNapp. Examples of how the selected BCTs were applied in SNapp are provided in Table 2.

### Step 2: Developing a Program Framework

#### Defining Objectives

It was determined that SNapp’s objectives were to (1) increase the number of steps users take per day to meet the recommended guidelines, equating to taking a minimum of 7000 to 8000 steps per day [43], and (2) promote the maintenance of regular walking when users meet these guidelines.

#### Describing Framework Components

##### Format of Feedback Delivery

Text messaging was considered a suitable format for feedback delivery. Text messages can deliver individually tailored PA support instantaneously and cost-effectively [56]. In addition, texting services are widespread in the Netherlands, with approximately 82% of adults aged 45 to 75 years exchanging text messages via messaging apps [57]. Furthermore, evidence from meta-analyses confirms that text message interventions produce positive changes in PA [56,58,59]. Hence, we decided that SNapp’s tailored feedback would be delivered through text messages.

##### Type of Feedback Delivery

Concerning the type of feedback that SNapp should provide, we strived to use three parameters to tailor coaching messages: (1) step count, (2) geolocation, and (3) BCT preferences. First, we decided that SNapp should be able to give feedback on step count, as this is consistent with the metrics of its objectives and is therefore helpful to comment on users’ progress. In addition, research on adults’ preferences for PA apps confirms that receiving feedback on performance is among the top-ranked BCTs [42,60].

Second, we determined that SNapp should offer contextually tailored prompts when users are near green spaces where they can walk, such as parks and walking trails. Research by Klasnja et al [61] has suggested that context-specific walking prompts can help individuals engage in bouts of walking throughout the day and are therefore a promising strategy to enhance the effectiveness of PA interventions. However, contextually tailored feedback has seldom been incorporated into PA apps, and more studies are needed to determine its efficacy [62]. Hence, we sought to fill this gap by incorporating contextually tailored walking prompts in SNapp.

Third, we chose to include users’ individual preferences for specific BCTs as our final tailoring parameter. Previous studies have examined the general evaluations of BCTs applied in PA apps by various populations [42,60] or analyzed the effectiveness of different BCTs in PA interventions [63]. Nevertheless, individual differences in BCT preferences have rarely been considered when tailoring app-based interventions. Users’ preferences regarding the BCTs applied in PA apps will likely play a role in how successfully a given app can stimulate behavior change. For example, some users may find that receiving prompts for goal setting or action planning is most helpful in improving their walking levels. In contrast, others may be more motivated by obtaining insights into the consequences of engaging in regular walking and ways of overcoming common barriers. Therefore, we opted to use BCT preferences to tailor SNapp’s coaching messages to individual users.

##### Frequency and Timing of Feedback Delivery

As SNapp aims to achieve sustained changes in daily walking behavior, we strived to develop a multiple-contact intervention in which tailored feedback is delivered daily. Meta-analyses have demonstrated that tailored interventions with multiple feedback points are more effective than single-contact interventions [64,65]. Following the study by Middelweerd et al [44], we determined that SNapp users should receive at least 3 daily coaching messages. Users should receive a message tailored to their BCT preferences each morning and evening. At midday, a message tailored to the step count should be delivered. Additional messages tailored to geolocation should be delivered whenever users are near green spaces where they can walk, with a maximum of 3 geolocation messages being sent daily and not more than once every 4 hours.

##### Data Collection Methods

As a first data collection method, we determined to imbed questions measuring users’ BCT preferences in a web-based baseline survey that would be completed before installing SNapp. In addition, we decided to rely on sensors in users’ smartphones to continuously measure the step count and geolocation via an app. Smartphone ownership has increased to approximately 93% among Dutch adults aged 45 to 75 years [57]. Therefore, we chose to use smartphone sensors for data collection instead of external hardware to eliminate the burden of wearing a separate tracker from the user, as perceived ease of use is an important determinant of willingness to use PA apps [66]. In addition, we did not want to create financial barriers to using SNapp by requiring the purchase of a separate tracker.

Evidence for the measurement accuracy of step counter apps is mixed. Some studies have demonstrated that apps can provide valid step counts [67-69], whereas others have found that they underestimate step counts, mainly because people do not always carry their phones on their person throughout the day [70-73]. Nevertheless, meta-analyses have indicated that app-based PA interventions can significantly increase the daily step count in adult populations [74-76]. Therefore, considering SNapp’s objective to observe improvements in daily walking behavior over time, rather than conducting absolute measurements of step count, we concluded that it would be adequate to use smartphone sensor functionalities to collect step count and geolocation data with an app.

### Step 3: Developing Tailoring Assessments

#### Developing Tailoring Assessment Questions

BCT preferences were assessed in a web-based baseline survey by asking users to indicate their stance toward receiving 10 types of coaching messages containing different BCTs on a 3-point scale (−1=negative, 0=neutral, and +1=positive). An example message was included for each message type (eg, “Messages that encourage me to plan when and where to go for a walk”). Multimedia Appendix 1 provides an overview of the BCT preferences measure.

#### Designing and Developing an App

**Figure 2:**
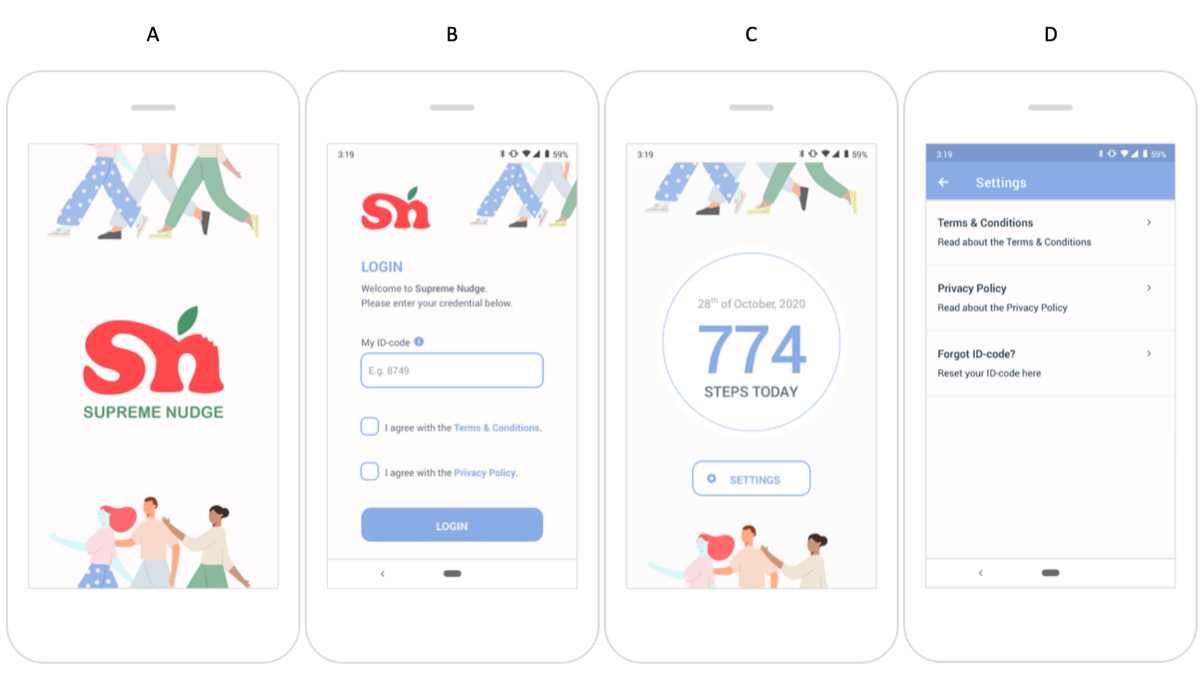
Screenshots: (A) launch screen, (B) log-in screen, (C) step count screen, and (D) settings screen.

On the basis of the set requirements, 2 separate native apps were developed for Android and iOS to allow the app to run smoothly on both operating systems. The developed app continuously quantifies the step count using the smartphone’s built-in pedometer or accelerometer. It is designed to detect steps when users carry their phone on their person, for example, when placed in one’s pants or coat pocket. In addition, to register geolocation, the app uses GPS data to compare the user’s current location with a database of preselected green spaces suitable for walking every 2 minutes. The app developer helped design the user interface, suggesting the layout, fonts, and graphic illustrations. Figure 2 shows the resulting app. It opens with a launch screen (Figure 2A). Next, users can log in by entering their user ID and agreeing with the terms and conditions and privacy policy (Figure 2B). Subsequently, they can view their daily step count (Figure 2C). Finally, users can access the terms and conditions and privacy policy or log out by clicking the settings button (Figure 2D).

### Step 4: Writing Tailored Messages

#### Creating a Tailored Message Database

A set of messages was written for each of the 3 tailoring parameters (ie, step count, geolocation, and BCT preferences). A consistent tone, format, and length were maintained for each message. The final message database contained 289 messages. Examples are provided in Table 2.

#### Testing Messages for Readability

The Common European Framework of Reference for Language [46] includes 6 reading levels (ie, A1, A2, B1, B2, C1, and C2), with A1 being the easiest and C2 the most difficult reading level. In the Netherlands, reading level B1 is recommended as appropriate for the general population [77]. Therefore, we tested whether the reading level of our messages was rated as B1 and adjusted them if necessary.

### Step 5: Automating the Tailoring Process

#### User Data Storage

Before users install SNapp, BCT preferences collected through the web-based baseline questionnaire are entered manually into the user data database (Figure 1). For each user, a BCT is rated with a score of 1 if users had a negative attitude toward it, a score of 5 if they felt neutral, and a score of 10 if they had a positive attitude toward it. Users’ first names are also entered manually into the database to allow addressing users by name in the coaching messages.

Every hour, the user database is updated with the user’s most recent step count data tracked by the app. The date, time of the latest step count update, step count level, and type of sensor used for tracking (ie, a pedometer or accelerometer) are stored in the database. To collect geolocation data, green spaces suitable for walking were preselected using Google Maps. Once a day, the app downloads the most recent version of this list of green spaces with their GPS coordinates and type (ie, a park, forest, or walking trail). Every 2 minutes, the device’s current location is compared with the internal database of green spaces. It is checked whether a user is within a 300-m radius from a green space by comparing the GPS coordinates of the user’s location with the coordinates of the preselected green spaces. A time stamp and the type of green space a user was near are stored for each observation.

#### Developing Tailoring Algorithms

SNapp’s 3 types of coaching messages are sent according to the following logical rules. First, a message tailored to step count is sent at a random time between 11:30 AM and 12:30 PM each day. Message selection is based on elimination, meaning irrelevant messages are filtered out from the complete message database to narrow down the selection. All messages containing feedback on step count are first selected from the database. Next, a subset of appropriate messages is selected depending on the users’ current performance levels (ie, ≤2000, 2001-6000, or ≥6001 steps). Finally, from the resulting subset, 1 message is randomly chosen to be sent. Personal values such as first name and the number of steps taken are inserted into the message to tailor it to the individual user.

Second, contextually tailored messages are sent whenever users are near the preselected green spaces. These messages are delivered not more than once every 4 hours between 8 AM and 8 PM. Depending on the type of green space users encounter (ie, a park, forest, or walking trail), the subgroup of messages that refers to this type is selected from the message database, from which one is randomly chosen to be delivered to the user. Users’ first names and the type of green space are inserted into the message to tailor its content to the individual user.

Third, to deliver messages tailored to BCT preferences, the system checks which messages are appropriate to send to the user every day at a random time between 8 AM and 9 AM and between 4 PM and 6 PM. First, all messages containing BCTs relevant to send given the day and time are collected. A subset of 4 messages is then selected from this list of relevant messages. To tailor messages to individual users, the probability of selecting a message for this subset is proportional to the preference score users have given the BCT included in the message. For example, a message containing a BCT with a positive preference score of 10 is 10 times more likely to be selected than a message containing a BCT with a negative preference score of 1. Finally, 1 of the 4 messages is randomly chosen to be sent to the user to increase the diversity of the delivered messages.

#### Creating a Communication Channel

**Figure 3:**
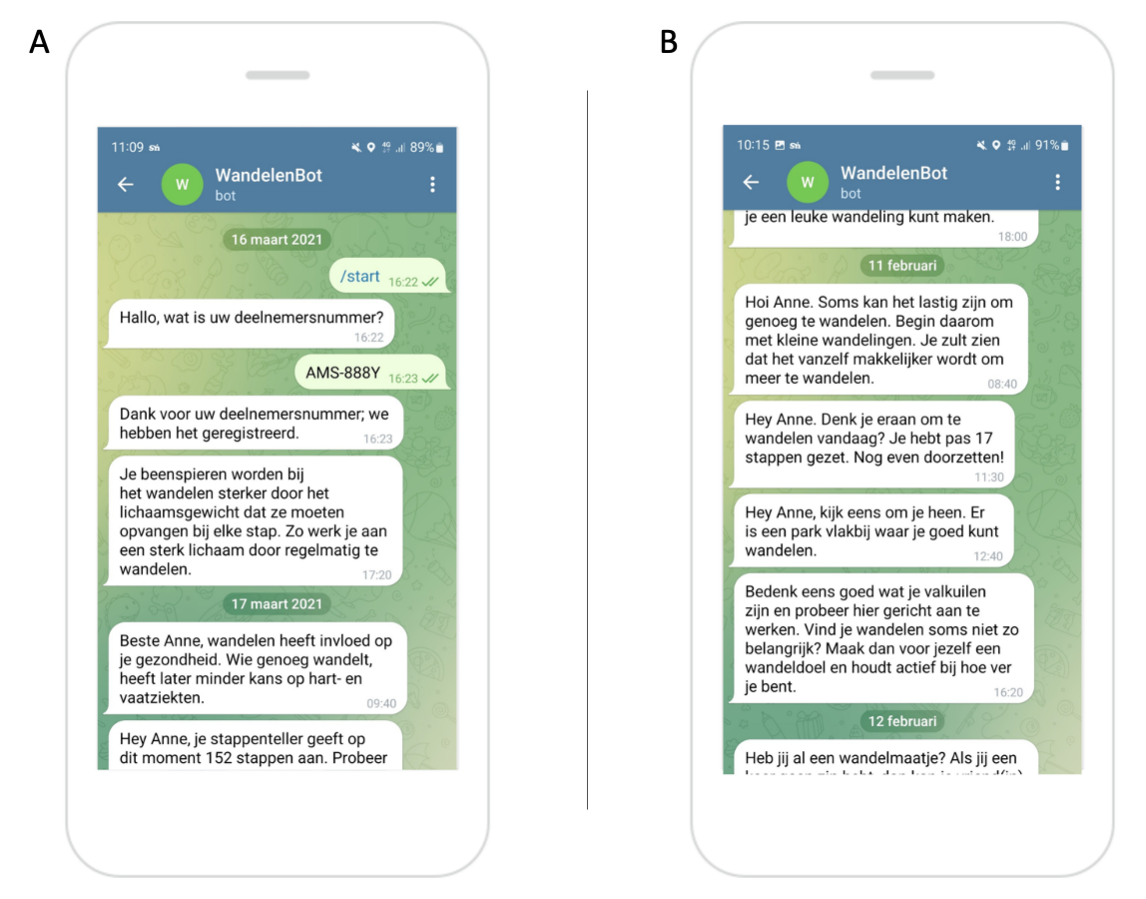
Screenshots of the (A) log-in procedure and (B) tailored coaching messages sent by the SNapp Telegram account.

To receive tailored messages, users should have Telegram installed on their smartphone and start a chat with the SNapp Telegram account. When starting this chat, users are asked to register with the same user ID they use to log in to our app, obviating the need to store personal data such as their telephone number or Telegram username (Figure 3A). Next, the entered user ID is checked for validity against the list of user IDs stored in the user data database. Users who enter an invalid user ID are asked to register again with the correct ID. After registering, the SNapp Telegram account sends users tailored coaching messages via push notifications without requiring user input (Figure 3B). Technically, it is possible for users to mute the SNapp Telegram account to no longer receive push notifications. However, users are not actively informed about this possibility.

### Step 6: Implementing and Evaluating the Program

The main feedback points that emerged during the pilot study interviews are described in the following paragraphs. First, the participants commented positively on the design of the app. They described it as colorful, cheerful, and nice looking. In addition, the participants appreciated the simple layout:

I like the illustrations. Cute figures and colors.

Very cheerful, and clear.

Second, most participants indicated that the app accurately tracked their daily step count. They predominantly carried their smartphones in their pants or coat pocket and reported that the app’s step counts corresponded with their walking behavior. Several participants also noticed no significant differences between the number of steps measured by our app and other PA apps that they used simultaneously:

I once compared the steps counted by the app and my fitness tracker for the same route. The app had counted some more steps, but it was only a small difference.

The number of steps is exactly the same as counted by another app I use.

However, a few participants reported technical issues related to step counting. For example, they mentioned that sometimes the app would not update to the correct date and reset the step count. Another technical problem was that no step count data were communicated to the database for some participants, although the app had successfully displayed the step count:

I noticed last week that it got stuck. For a few days, it got stuck on December 9. It did not follow up to the next day.

Third, most participants thought the message content was acceptable. A few participants indicated that SNapp had stimulated them to go for an extra walk because it had made them more aware of their walking behavior. Some mentioned that they thought the coaching messages would be most helpful for inactive individuals:

I don’t want to receive disappointing messages, so it did make me go for an extra walk to prevent that from happening.

Look, if you are not very active and you need a lot of encouragement, I think it’s nice. The more personal it gets, the better. If you really get the feeling that someone is watching and then says: great, you’ve done well.

Finally, participants were interested in including more functionalities. For example, a few participants indicated that they did not always carry their smartphones with them throughout the day. They were disappointed that the app was then unable to measure steps and suggested that it would be helpful if it could be used in combination with wearables. Additional suggestions included tracking the duration of walks, walking speed, burned calories, the number of kilometers, and walking routes. Finally, participants mentioned that it would be helpful if the app could display their daily step count over the past week to obtain an overview of their progress:

Sometimes I wear clothes without pockets. And then I don’t always have my phone with me. I also don’t want to be forced to have my phone with me all the time. A fitness tracker could then still record everything.

You can’t see what you’ve been doing the past few days. That’s something I miss. But you also do not see where you walked. I think I would find that very interesting. Or kilometers. I’ve walked so much, so many kilometers.

On the basis of these findings, final refinements were made to solve the identified technical issues. It was subsequently concluded that SNapp’s design, technical functioning, and coaching content were acceptable. Owing to time and budget constraints, it was impossible to include the additional functionalities suggested by the participants. However, these suggestions are worth considering for future updates or similar app-based interventions. After resolving the engineering bugs, SNapp’s development process was completed.

## Discussion

### Principal Findings

The purpose of this study was to present a detailed description of SNapp's theoretical background and stepwise development process using the program planning model by Kreuter et al [34]. SNapp is an app-based intervention that aims to coach adults of low SEP to increase activity levels by offering tailored support targeting walking behavior. Within SNapp, smartphone sensor functionalities are combined with theory-based BCTs to provide coaching messages that are individually tailored using step count, geolocation, and BCT preferences data.

Among the strengths of SNapp are its grounding in health behavior theory and the use of evidence-based strategies throughout the development process. In addition, SNapp goes beyond existing PA apps and app-based interventions by offering individually tailored content in line with users’ BCT preferences. For example, users who favor action planning and self-monitoring prompts to motivate them are more likely to receive messages that include these BCTs. In contrast, others may prefer and thus receive other coaching content, such as goal setting or barrier identification strategies. We expect this to improve the personal relevance of SNapp’s coaching messages, resulting in higher motivation to follow up on them. Another novel component of SNapp is the use of geofencing techniques to send contextually tailored suggestions for PA when users are near green spaces where they can walk, which allows SNapp to target users with coaching messages at the right time and place.

Certain limitations of this study can be addressed in future work. First, we are currently extending SNapp by implementing machine learning techniques to make delivered coaching content more dynamically tailored based on changes in users’ behavior, thereby increasing its efficacy. For example, integrating artificial intelligence techniques that allow SNapp to learn which coaching content is particularly good at triggering walking behavior for individual users within specific contexts can help improve the effectiveness of delivered coaching messages [78,79]. Second, we feel that future iterations may benefit from including more conditional factors (eg, weather, seasonal conditions, or physical environment attributes) to improve the context specificity and relevance of suggestions for walking. Third, a helpful avenue for future updates would be the inclusion of more functionalities within SNapp. For example, integrating a chatbot into the system that can interpret textual input would allow users to interact with SNapp, thereby improving the user experience [80]. In addition, as the findings from our pilot study suggest, possibilities to synchronize SNapp with wearables and track other types of walking data apart from daily step count are worth considering.

By conducting an exploratory qualitative pilot study that allowed participants to use SNapp in their personal environments over the course of 2 weeks, we collected valuable feedback on its basic technical functioning and confirmed the acceptability of its design and content for users. Prior research has also underlined the benefits of qualitative methods for gaining beneficial insights into the usability of health apps and interventions [81,82]. However, the limitations of the pilot study were its small study sample that did not specifically include SNapp’s target population of adults of low SEP and the short duration of the study. Therefore, this work will be extended in future research to explore the effectiveness and wider applicability of SNapp. The final version of SNapp described in this paper was implemented in a real-life parallel cluster-randomized controlled trial that is being conducted with the aim of improving lifestyle behaviors in populations of low SEP. Participants of this trial are recruited from socially disadvantaged areas (based on below-average postal code SEP scores [83]) and will use SNapp for a period of 6 to 12 months. The protocol of this trial has been described in detail elsewhere [31]. Findings regarding short- and long-term effects, mediating mechanisms, and moderators of intervention effects are forthcoming to ensure a critical evaluation of SNapp’s efficacy.

### Conclusions

In this paper, we presented the stepwise development process of SNapp, guided by the program planning model of Kreuter et al [34]. The resulting app-based intervention aims to promote walking among adults of low SEP by offering coaching messages that are individually tailored using step count, geolocation, and BCT preferences data. SNapp has a solid theoretical grounding, is privacy aware, and is supported by user feedback collected in a pilot study. In future work, we will evaluate the short- and long-term effects of SNapp based on the randomized controlled trial results in which SNapp is currently implemented.

## Appendix

Multimedia Appendix 1 Behavior change technique preferences measure.

## Abbreviations

BCT: behavior change technique
CVD: cardiovascular disease
PA: physical activity
SCT: social cognitive theory
SEP: socioeconomic position

## Data Availability

The data sets generated and analyzed during this study are available from the corresponding author on reasonable request.
