# Supplementary material for: SNapp, a Tailored Smartphone App Intervention to Promote Walking in Adults of Low Socioeconomic Position: Development and Qualitative Pilot Study
Source: JMIR Form Res. 2023 Apr 17;7:e40851. doi: 10.2196/40851 (PMC10152336; doi:10.2196/40851)
Supplement: Multimedia Appendix 1 [file formative_v7i1e40851_app1.docx]

**BCT Preferences Measure**

A walking app can stimulate you to walk (more) by means of personal messages. We are curious which personal messages would appeal to you most in a walking app. Please indicate to what extent you would feel positive or negative about receiving the messages below. You can answer with a number between -1 and +1, where -1 stands for "Negative", 0 stands for "Neutral" and +1 stands for "Positive".

| Type of message | Example |
| --- | --- |
| 1. Messages with information about the (health) consequences of walking. | “Walking is good for your health. Walking every day has a positive influence on your weight, heart, and blood pressure.” |
| 1. Messages that provide insight into the walking behavior of others. | “More than three quarters of all SNapp users managed to walk for at least half an hour every day last week!” |
| 1. Messages that encourage me to plan when and where to go for a walk. | “To walk enough, it helps to plan when and where you will go for a walk in the coming days. Write it down in your calendar in advance.” |
| 1. Messages that stimulate me to keep track of whether I am taking enough steps per day. | “Try to keep track of how much you walk every day. This can help you meet your walking goal each week. Our app shows how many steps you’ve already taken today.” |
| 1. Messages that give me tips on how to get support from others for walking. | “By regularly going for a walk with someone else, it is easier to keep it up. So, try asking if someone wants to go for a walk with you.” |
| 1. Messages that encourage me to set challenging walking goals for myself. | “Do you already know what your walking goal will be for this week? Think of a new challenge for yourself. For example, you can come up with a goal for the number of steps you want to take each day.” |
| 1. Messages that give me tips on how to overcome barriers to walking. | “Does it sometimes feel like you don't have time to walk? A walk doesn't have to be long. You can also take short walks at different times of the day.” |
| 1. Messages that ask me to evaluate how satisfied I am with my walking behavior. | “Check with yourself whether you are satisfied with how often you have walked lately. What are you proud of and what could be improved?” |
| 1. Messages with encouragements to improve my walking behavior. | "Keep it up! Remember to walk enough today. You can do it." |
| 1. Messages that provide insight into what others think of my walking behavior. | "Others around you will surely be proud of you if you manage to keep walking regularly." |
